# Association between mental health and academic performance among university undergraduates: The interacting role of lifestyle behaviors

**DOI:** 10.1002/mpr.1938

**Published:** 2022-09-10

**Authors:** Tianshu Chu, Xin Liu, Shigemi Takayanagi, Tomoko Matsushita, Hiro Kishimoto

**Affiliations:** ^1^ Department of Behavior and Health Sciences Graduate School of Human‐Environment Studies Kyushu University Fukuoka Japan; ^2^ Center for Health Sciences and Counseling Kyushu University Fukuoka Japan; ^3^ Faculty of Arts and Science Kyushu University Fukuoka Japan

**Keywords:** impaired mental health status, lifestyle behavior, poor academic performance, prospective study, undergraduate

## Abstract

**Objectives:**

Impaired mental health status tends to be associated with poor academic performance, but few prospective studies have examined the association between mental health and academic performance among undergraduates while considering the interacting roles of multiple lifestyle behaviors.

**Participants and Methods:**

A total of 1823 Japanese undergraduate students (67% men) were followed up for 4 years. Their mental health status was measured by the six‐item Kessler Psychological Distress Scale (K6). We defined poor academic performance as a grade point average (GPA) <2.0. Cox proportional hazards models were used to determine the relationship between the students' mental health status and the incident risk of poor academic performance.

**Results:**

Our analyses revealed that impaired mental health status in the first semester of university study significantly predicted an increased incident risk of poor academic performance during the overall undergraduate period. This association remained significant when the health lifestyle behaviors were adjusted, and the hazard ratio (95% confidence interval) for poor academic performance was 1.62 (1.18–2.23). This significant association disappeared in the low‐lifestyle‐behavior‐risk group.

**Conclusion:**

Impaired mental health status in the first semester significantly predicts an increased incident risk of poor academic performance during the undergraduate period.

## 1 INTRODUCTION

Mental health issues among undergraduate university students are increasing. A systematic review estimated that 12%–46% of undergraduates are distressed by mental health issues (Harrer et al., 2019). Although impaired mental health is becoming a critical public health issue, the World Health Organization World Mental Health Surveys in 2016 revealed that just one‐fifth of students have sought help and adequate treatment for mental health issues (Auerbach et al., 2016). A tendency for a relationship between impaired mental health status and poor academic performance in undergraduate students was reported (Castaneda et al., 2008); however, most of the relevant research has focused on the cross‐sectional relationship between an impaired mental health status and poor academic performance (Castaneda et al., 2008; Wickersham et al., 2021).

It is unknown whether having an impaired mental health status could predict the incident risk of poor academic performance during the overall undergraduate period. In addition, most of the studies published to date collected the students' self‐reported academic performance data by questionnaire (Tembo et al., 2017). It is necessary to use the objective academic performance data obtained from the university affairs divisions.

Moreover, health lifestyle behavior is a critical factor when discussing the association between impaired mental health status and poor academic performance. University undergraduate students represent a specific population that is transitioning from adolescence to adulthood, experiencing various changes. To cope with the challenges in their new university environment, some students are more likely to experience difficulty managing lifestyle behaviors such as eating/skipping breakfast or sleeping late, which have been suggested to be associated with the poor academic performance (Kayaba et al., 2021). Unhealthy lifestyle behaviors (e.g., smoking habit, lack of exercise) are also correlated with an impaired mental health status (Doom et al., 2013). However, few studies have provided any insight into the association between mental health and academic performance among undergraduates while considering the interacting role of multiple lifestyle behaviors. For example, it has not been clarified whether students with poor mental health have a low risk of poor academic performance if their lifestyle is healthy.

The primary purpose of the present study was to investigate the association between impaired mental health status and poor academic performance among Japanese undergraduate students. The secondary purpose was to determine the influence of healthy lifestyle behaviors on the association between mental health status and poor academic performance.

## 2 METHODS

### 2.1 Study design

This study used data provided by the Enhancement of K‐University Students Intelligence (EQUSITE) survey, which was performed from 2011 to 2015 among Japanese university students. The details of the EQUSITE survey have been described (Supartini et al., 2016). We carried out a population‐based prospective study focusing on the association between impaired mental health status at the students' first semester and the incident risk of poor academic performance during a 4‐year follow‐up period.

### 2.2 Participants and procedures

**FIGURE 1:**
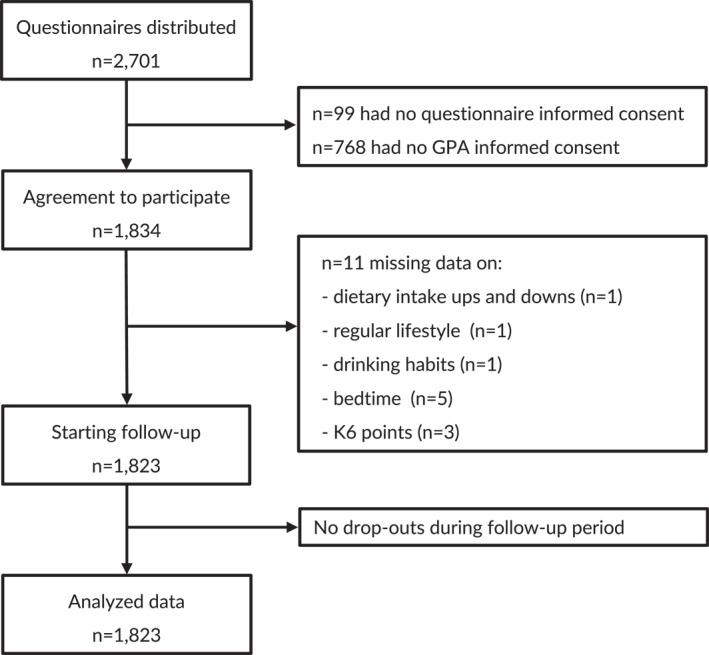
Flowchart of the participant selection

The participants were 2701 undergraduate students who were newly enrolled at K. University in April 2011. The baseline survey was administered in May and June of 2011. The students' first semester's academic performance data were obtained from their final examination conducted in September 2011. All of the students' academic performance data for the undergraduate period until their last semester were provided by the university administration in April 2015. As shown in Figure 1, a final total of 1823 participants were included in the present analyses.

### 2.3 Academic performance

The students' academic performance was based on their grade point average (GPA) assigned on a scale from 0.0 to 4.0 scores, with lower GPA scores indicating poorer academic performance. Each student's GPA score was calculated as the credit‐weighted sum of his or her grades for all courses divided by the total number of course credits. There is no value was defined as the normal cut‐off value of GPA scores, but earlier studies used the means of GPA scores (Awadalla et al., 2020; BlackDeer et al., 2021). In the present study, we used a GPA <2.0 score that was newly identified during the 4‐year follow‐up period as the definition of poor academic performance.

### 2.4 Mental health

The students' mental health status was measured by the six‐item Kessler Psychological Distress Scale (K6). Each item uses a five‐point Likert scale ranging from 0 to 4 points. The possible number of total points ranges from 0 to 24 points, with a greater number of points indicating a higher‐level impaired mental health status (Kessler et al., 2002). The reliability and validity of the K6 scale for screening undergraduate students' mental health status have been confirmed (Kang et al., 2015; Stallman, 2010). Based on previous evidence, we divided the present participants into three K6 groups (using 5 and 13 points as the cut‐off points) to monitor the levels of the students' mental health status (Arima et al., 2020; Furukawa et al., 2008; Sato, Watt et al., 2020).

### 2.5 Correlates

The students' self‐reported answers on questionnaires at the 2011 baseline survey described their health lifestyle behaviors as the correlates. The primary health lifestyle behavior variables included weekday study time and game time, drinking habit, exercise frequency, breakfast habit, dietary intake ups and downs, wake time, all of which were verified as risk factors of poor academic performance in part of the present cohort population (Hutchesson et al., 2021). The health lifestyle behavior risk scores calculated using the above variables ranged from −3 to 11 points, in which higher risk scores indicate unhealthier lifestyle behaviors. We used the median value of the students' risk scores to divide them into the healthy lifestyle behavior group (i.e., the low score group) and the unhealthy lifestyle behavior group (the high score group).

### 2.6 Statistical analyses

We first used the Cochran‐Armitage trend test and Jonckheere‐Terpstra trend test to calculate the trends of healthy lifestyle behaviors according to the three K6 groups. We then calculated the crude hazard ratios (HRs) and multi‐variable adjusted HRs with 95% confidence intervals (CIs) for the incident risk of poor academic performance according to K6 groups, using multiple Cox proportional hazard models. The same analysis protocol was repeated to explore the association between the three K6 groups and the incident risk of poor academic performance in the different healthy lifestyle behavior groups, as appropriate. A two‐sided *p*‐value <0.05 was considered significant. All analyses were completed using SAS ver. 9.4 (SAS Institute, Cary, NC, USA). The computations were carried out using the computer resources offered under‐General Projects by the Research Institute for Information Technology, K. University.

### 2.7 Ethical considerations

The Ethics Committee of the Institutional Review Board of Health Science, K. University approved this study (Approval ID, HIS‐2011‐02; approval date, May 11, 2011).

## 3 RESULTS

During the four‐year follow‐up period, with no participants dropping out, all 1823 participants were included in the final analysis. The trends of healthy lifestyle behavior variables at baseline according to the three K6 groups are shown in Table 1. According to the K6 groups from low to high points, most of the variables showed significantly increased trends within unhealthy lifestyle behaviors.

**TABLE 1:** Characteristics of the university undergraduate students who participated in the study

	K6 groups			*p*‐value for trend
	0–4 (*n* = 1010)	5–12 (*n* = 697)	13–24 (*n* = 116)	
K6, points	1.6 ± 1.4	7.7 ± 2.3	15.6 ± 2.6	<0.0001
Men	723 (71.6)	414 (59.4)	83 (71.6)	0.001
Health behaviors, score				
Men (*n* = 1220)	9.3 ± 6.8	11.1 ± 7.3	13.7 ± 7.5	<0.0001
Women (*n* = 603)	2.5 ± 6.1	3.7 ± 6.0	4.8 ± 5.8	0.002
Age, years	18.3 ± 0.7	18.3 ± 0.6	18.4 ± 0.6	0.06
Regular lifestyle				<0.0001
Yes	605 (59.9)	331 (47.5)	43 (37.1)	
No	405 (40.1)	366 (52.5)	73 (62.9)	
Weekday study time				0.04
≥1 h	391 (38.7)	238 (34.1)	38 (32.8)	
<1 h	619 (61.3)	459 (65.9)	78 (67.2)	
Weekday video game time				0.04
<1 h	877 (86.8)	586 (84.1)	94 (81.0)	
≥1 h	133 (13.2)	111 (15.9)	22 (19.0)	
Drinking habit				0.02
Never	637 (63.1)	414 (59.4)	62 (53.5)	
More than once a week	373 (36.9)	283 (40.6)	54 (46.5)	
Exercise habit				0.13
Almost every day	139 (13.8)	78 (11.2)	13 (11.2)	
Sometimes or never	871 (86.2)	619 (88.8)	103 (88.8)	
Breakfast habit				0.04
Almost every day	792 (78.4)	533 (76.5)	84 (72.4)	
Sometimes	135 (13.4)	99 (14.2)	14 (12.1)	
Never	83 (8.2)	65 (9.3)	18 (15.5)	
Dietary intake ups and downs				<0.0001
No	766 (75.8)	429 (61.6)	49 (42.2)	
Yes	244 (24.2)	268 (38.5)	67 (57.8)	
Waking time				0.31
07:00 or earlier	695 (68.8)	478 (68.6)	72 (62.1)	
Later than 07:00	315 (31.2)	219 (31.4)	44 (37.9)	

*Note*: Data are mean (standard deviation) or *n* (%). Statistical significance was based on Cochran‐Armitage trend tests or Jonckheere‐Terpstra trend tests, as appropriate.

**FIGURE 2:**
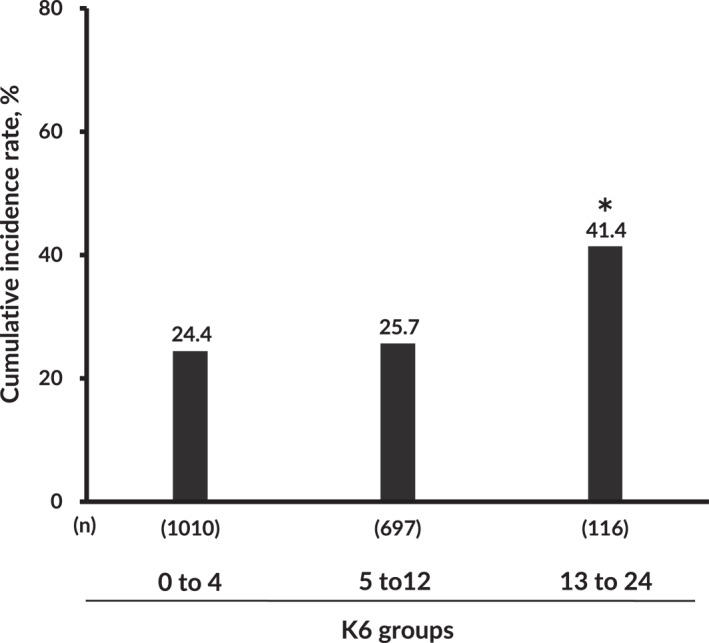
The cumulative incidence rate of poor academic performance (GPA <2.0) during the 4 years of university study. *p < 0.0001 versus 0–4‐point group, *p* for trend <0.01

Figure 2 illustrates the crude cumulative incidences of poor academic performance classified according to the K6 groups. A continuous increase in the crude cumulative incidences of poor academic performance starts at 24.4% in the 0–4‐point K6 group, 25.7% in the 5–12‐point K6 group, and 41.4% in the 13–24‐point K6 group. The incident risk of poor academic performance in the 13–24‐point K6 group was significantly higher than that in the 0–4‐point K6 group.

The data clarifying the incident risk of poor academic performance classified according to the K6 groups are provided in Table 2. In the crude model, the incident risk of poor academic performance in the 13–24‐point K6 group was significantly higher than that in the 0–4‐point K6 group. In the multivariable adjusted model, after adjusting for the students' sex, major field of study, and healthy lifestyle behavior risk scores, the incident risk of poor academic performance in the 13–24‐point K6 group was also significantly higher than that in the 0–4‐point K6 group, and the HR (95%CI) for poor academic performance was 1.62 (1.18–2.23). In addition, the crude HR (95%CI) and multivariable adjusted HR (95%CI) for poor academic performance with one‐point increases in the number of K6 points were 1.04 (1.02–1.06) and 1.03 (1.01–1.05), respectively.

**TABLE 2:** Association between K6 groups and the incident risk of poor academic performance during the 4‐year follow‐up

K6 groups	No. of events/participants	Crude HR (95%CI)	*p*‐value	Multi‐variable adjusted HR (95%CI)	*p*‐value
0–4	246/1010	1.00 (ref.)		1.00 (ref.)	
5–12	179/697	1.16 (0.95–1.41)	0.14	1.11 (0.91–1.35)	0.31
13–24	48/116	2.07 (1.51–2.83)	<0.0001	1.62 (1.18–2.23)	0.003
Per 1‐point increase			0.0001		0.01

*Note*: Adjusted for sex, major field of study, and lifestyle behavior risk scores.

Abbreviations: CI, confidence interval; HR, hazard ratio.

Table 3 explains the associations between the K6 groups and the incident risk of poor academic performance in the different healthy lifestyle behavior groups. In only the unhealthy lifestyle behavior group (i.e., the high score group), the incident risk of poor academic performance in the highest K6 group (13–24 points) remained significantly higher than that in the 0–4‐point K6 group regardless of whether sex and major field of study were adjusted.

**TABLE 3:** Association between K6 groups and the incident risk of poor academic performance during 4 years by lifestyle behavior risk scores

K6 groups	No. of events/participants	Crude HR (95%CI)	*p*‐value	Multi‐variable adjusted HR (95%CI)	*p*‐value
**Low lifestyle behavior risk scores**					
0–4	89/563	1.00 (ref.)		1.00 (ref.)	
5–12	54/357	1.08 (0.77–1.53)	0.64	1.15 (0.81–1.61)	0.44
13–24	11/49	1.80 (0.96–3.39)	0.07	1.76 (0.94–3.31)	0.08
Per 1‐point increase			0.11		0.08
**High lifestyle behavior risk scores**					
0–4	157/447	1.00 (ref.)		1.00 (ref.)	
5–12	125/340	1.13 (0.89–1.44)	0.30	1.20 (0.95–1.53)	0.13
13–24	37/67	1.96 (1.36–2.82)	0.0003	1.83 (1.27–2.64)	0.001
Per 1‐point increase			0.003		0.003

*Note*: The groups of low/high lifestyle behavior risk scores were divided by the health behavior scores' median value. Adjusted for sex and major field of study.

**FIGURE 3:**
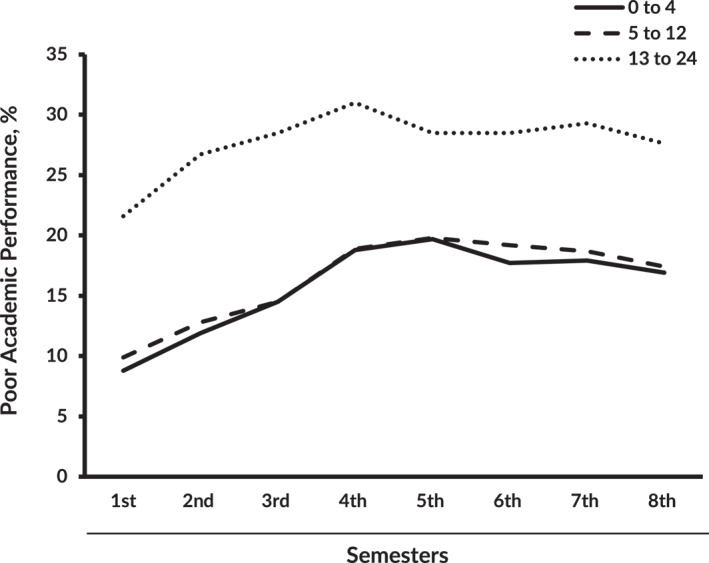
The percentage of students with poor academic performance (GPA <2.0) in each semester

Figure 3 depicts the distribution of poor academic performance (GPA score <2.0) in each semester. The percentage of poor academic performance shows a substantial increase from the third semester to the fourth semester.

## 4 DISCUSSION

The results of this prospective study demonstrate that impaired mental health status in the first semester of university studies significantly predicted an increased incident risk of poor academic performance among undergraduates during their overall undergraduate period. This association remained significant even with adjustment for health lifestyle behaviors. However, the significant association between impaired mental health status and the increased incident risk of poor academic performance disappeared in the group of students with a low lifestyle behavior risk.

A study of 1530 Canadian undergraduate students revealed that impaired mental health status in the first semester tended to account for lower GPAs at the end of the second semester (Duffy et al., 2020). That finding was based on a 1‐year follow‐up duration, whereas our study collected the academic performance data until the last semester of the 4‐year undergraduate period. Our analyses may thus have avoided the potential reverse causation bias that can occur during a short follow‐up period.

The results of our analyses demonstrated that the positive association between impaired mental health status and an increased risk of poor academic performance remained even with adjustment for the students' health lifestyle behavior risk scores. This suggests that a university student's mental health status is directly related to his/her future academic performance. Other research groups have proposed several mechanisms regarding this association. For example, there was a study reported that aspects of an impaired mental health status such as depressive or anxious symptoms tend to be associated with lower academic self‐efficacy, learning ability, and learning motivation, which might influence students' study progress and final academic outcomes (Grotan et al., 2019). Another study suggested that impaired mental health status can lead to negative emotions in daily life, affecting an individual's learning behavior, motivation, and task completion (Flueckiger et al., 2014). Other factors (such as social support) that we did not examine in the present study might be ‘hiding behind’ and affect the association between mental health status and the incident risk of poor academic performance (Rothon et al., 2011).

Health lifestyle behaviors may not be strictly related to either mental health status or academic performance. Interestingly, in the present study, the health lifestyle behavior risk score and K6 points showed a weak significant positive association (Pearson correlation coefficient: 0.13, *p*‐value <0.05), which suggested that the students classified in the low lifestyle behavior risk group (i.e., the healthy lifestyle behavior group) may have impaired mental health status. We thus also investigated whether being in the healthy lifestyle risk group moderated the association between mental health status and the incident risk of poor academic performance. The data in Table 3 show that the significant association disappeared in the low lifestyle behavior risk group, which implies that within this risk group, some healthy lifestyle behaviors (such as eating breakfast almost every day, being without dietary intake ups and downs, and an earlier waking time) weakened the positive association between impaired mental health and the incident risk of poor academic performance. The lower number of students in this group (*n* = 49 vs. 357 and 563 in the other groups; Table 3) may also have contributed to the lower statistical power. Our results thus indicate that even if a student is classified as having an impaired mental health status, the incident risk of poor academic performance may not increase significantly if the student maintains more healthy lifestyle behaviors.

Our findings also draw attention to the Health Sciences and Counseling division of the university that the students were attending; this division is essential to attempts to determine and improve students' unhealthy lifestyle behaviors. It is might optimal for students to lessen their risk of poor academic performance at an early stage by seeking advice from on‐campus counselors.

The results of this study strengthen the association between impaired mental health status and poor academic performance, as shown by a large sample of undergraduate students in a 4‐year follow‐up prospective study. Our findings are based on the objective academic performance data obtained from the university affairs division, which can be expected to be accurate. Nonetheless, some study limitations should be noted. First, although the K6 scale is widely used to estimate individuals' mental health status and the specificity of depression and anxiety, the K6 scale cannot be used for clinical diagnoses. Second, we did not collect the students' secondary‐school academic performance data, although this parameter might be a confounder variable that could have affected the students' academic performance during the undergraduate period. Finally, we defined the cut‐off value for poor academic performance based on a single university's regulation, and this cut‐off might not be applicable at other universities or in different countries.

## 5 CONCLUSION

Undergraduate students' impaired mental health status in the first semester significantly predicted an increased risk of poor academic performance during the undergraduate period. The significant association between impaired mental health status and the poor academic performance disappeared in the students with a low lifestyle behavior risk.

## AUTHOR CONTRIBUTORS

Tianshu Chu contributed to the study conception, design, formal statistical analyses, interpretation, and drafting of the manuscript. Xin Liu contributed to the data interpretation and reviewed the analysis and the manuscript. Shigemi Takayanagi and Tomoko Matsushita contributed to the data preparation and interpretation. Hiro Kishimoto as the corresponding author conceptualized and supervised the entire study, was responsible for obtaining funding, provided guidance on methodology, performed the statistical analyses, interpreted the results, and critically reviewed the manuscript. Hiro Kishimoto and Tianshu Chu accessed and verified all of the data. All authors had final responsibility for the decision to submit the manuscript for publication.

## CONFLICTS OF INTEREST

We have no conflicts of interest.

## Data Availability

The data used in this study are not publicly available, due to privacy considerations.
